# NHBA is processed by kallikrein from human saliva

**DOI:** 10.1371/journal.pone.0203234

**Published:** 2019-08-01

**Authors:** Elisa Pantano, Sara Marchi, Massimiliano Biagini, Martina Di Fede, Vincenzo Nardi Dei, Silvia Rossi Paccani, Mariagrazia Pizza, Elena Cartocci

**Affiliations:** 1 GSK, Siena, Italy; 2 GSK, Wavre, Belgium; Instituto Butantan, BRAZIL

## Abstract

Neisserial Heparin Binding Antigen (NHBA) is a surface-exposed lipoprotein of *Neisseria meningitidis* and a component of the Bexsero vaccine. NHBA is characterized by the presence of a highly conserved Arg-rich region involved in binding to heparin and heparan sulphate proteoglycans present on the surface of host epithelial cells, suggesting a possible role of NHBA during *N*. *meningitidis* colonization. NHBA has been shown to be cleaved by the meningococcal protease NalP and by human lactoferrin (hLF), a host protease presents in different body fluids (saliva, breast milk and serum). Cleavage occurs upstream or downstream the Arg-rich region. Since the human nasopharynx is the only known reservoir of infection, we further investigated the susceptibility of NHBA to human proteases present in the saliva to assess whether proteolytic cleavage could happen during the initial steps of colonization. Here we show that human saliva proteolytically cleaves NHBA, and identified human kallikrein 1 (hK1), a serine protease, as responsible for this cleavage. Kallikrein-related peptidases (KLKs) have a distinct domain structure and exist as a family of 15 genes which are differentially expressed in many tissues and in the central nervous system. They are present in plasma, lymph, urine, saliva, pancreatic juices, and other body fluids where they catalyze the proteolysis of several human proteins. Here we report the characterization of NHBA cleavage by the tissue kallikrein, expressed in saliva and the identification of the cleavage site on NHBA both, as recombinant protein or as native protein, when expressed on live bacteria. Overall, these findings provide new insights on NHBA as target of host proteases, highlights thepotential role of NHBA in the *Neisseria meningitidis* nasopharyngeal colonization, and of kallikrein as a defensive agent against meningococcal infection.

## Introduction

*Neisseria meningitidis* is a pathogenic, encapsulated, aerobic gram-negative diplococcus, member of the *Neisseriae* family. It is an obligate commensal in man and colonizes the nasopharyngeal mucosa without affecting the host, a phenomenon known as carriage recognized since 1890 [1] [2] [3]. In nonepidemic settings, approximately 10% of healthy individuals at any time carry *N*. *meningitidis* in the oropharyngeal tract. Transmission of bacteria occurs via direct contact or through dispersion of respiratory droplets from a healthy carrier or an infected person to a susceptible individual. Although often protected by a polysaccharide capsule, meningococci are particularly sensitive to desiccation; thus, spread from one individual to another necessitates close contact [4] [5]. In fact, closed or semi-closed settings, such as residential schools and military recruit camps, facilitate meningococcal transmission resulting in a dramatic increase of carriage rate, which may approach 100%.

Occasionally, shortly after the onset of colonization, *N*. *meningitidis* penetrates the mucosal membrane and enters the bloodstream, causing various forms of disease [6]. The most common clinical presentations of invasive infections include meningitis and severe sepsis with an often fatal outcome [7]; other diseases, such as septic arthritis, pneumonia, purulent pericarditis, conjunctivitis, otitis, sinusitis and urethritis, occur more rarely [6].

Meningococcal serogroups B and C are responsible for the majority of meningitidis cases in Europe, Y serogroup is relevant in the United States [8], whereas African epidemics are mainly attributable to the A strain. While effective glycoconjugate vaccines based on capsular polysaccharides against serogroups A, C, W and Y have been available for many years, different strategies have been required for developing a vaccine against the serogroup B since its capsular polysaccharide is structurally identical to a component of the human fetal neural cell-adhesion molecule [9]. In 2000, serogroup B genome mining and subsequent reverse vaccinology analysis led to the discovery of three main protein antigens. These proteins, either as single antigens or as fusion proteins, were formulated with the Outer Membrane Vesicles from a New Zealand outbreak strain (NZ98/254) shown to be protective in an immunization campaign in New Zealand [10]. Nowadays, Bexsero, is a multicomponent vaccine against *Neisseria meningitidis* serogroup B [11].

Neisseral Heparin Binding Antigen (NHBA), previously named as GNA2132 (Genome-derived Neisseria Antigen 2132), is one of the three main protein antigens of the Bexsero vaccine. NHBA is a surfaced-exposed lipoprotein, the corresponding *nhba gene* is ubiquitous in meningococcal strains of all different serogroups and has also been found in several other *Neisseria* species, including *N*. *lactamica*, *N*. *polysaccharea* and *N*. *Flavescens* [12]. Recent studies on NHBA have shown that this protein is implicated in different steps of meningococcal pathogenesis, including bacterial adhesion to epithelial cells [13], biofilm formation [14], bacterial survival in the blood, and vascular leakage [15]. It is also able to bind heparin *in vitro* through an Arg-rich region [12] and this ability may point to a role of NHBA in the protection of unencapsulated meningococci against complement [15].

The primary amino-acid sequence of NHBA, from strain 2996, comprises approximately 450 residues and its structure can be divided into two main domains: an N-terminal region (residues 1 to ~230), marked as an intrinsically unfolded region, and a C-terminal region, whose structure, organized in β-barrel (residues 305–426) with high thermal stability, has been solved by NMR spectroscopy and more recently by X ray crystallography [16] [17]. A highly conserved Arg-rich motif, responsible for NHBA binding to heparin and heparan sulfate proteoglycans [15] [12], (residues 235–245) is located between the N- and C-terminal regions [11] [16]. It has been reported [12] that NHBA can be processed upstream or downstream of the Arg-rich region by the bacterial NalP protease and by human lactoferrin (hLF), respectively. Since hLF is a protease present in various body fluids, including saliva, we hypothesized that NHBA protein could be cleaved by proteases contained in the saliva during the initial step of *N*. *meningitidis* colonization of the nasopharyngeal mucosa. Here we show that NHBA is proteolyzed during incubation with saliva and identified kallikrein 1 (hK1) as responsible for the cleavage. Kallikreins are a family of serine proteases, with distinct domain structure which exist as a family of 15 genes differentially expressed in many tissues and in the central nervous system [18]. Kallikreins are present in lymph, urine, saliva, pancreatic juices, and other body fluids and are able to catalyze the proteolysis of kininogens and release kinins [19] [20]. The cleavage sites of hK1 correspond to the amino-acid pair Met-Lys and Arg-Ser in low molecular weight kininogen (LK) polypeptide precursor, while it occurs after the Phe–Phe pair in kallistatin and somatostatin [21].

Here we show that NHBA fragments generated by hK1 cleavage and characterized by mass spectrometry are identical to the NHBA fragments generated by human lactoferrin proteolytic cleavage. On live bacteria, NHBA cleavage by hK1, present in saliva, and human plasma kallikrein, PKa, results in the release of the C-fragment of approx 100 residues in the culture supernatant.

## Materials and methods

### SDS-PAGE and immunodetection analysis

Proteins were separated by SDS-PAGE using 4–12% or 12% polyacrylamide NuPAGE Bis-Tris Precast Gels (Invitrogen). For SDS-PAGE analysis, gels were stained with SimplyBlue Safe Stain (Invitrogen). For immunodetection analysis, 1 ml of culture medium supernatant was recovered, filtered trough a 0.22 μm cut-off membrane and precipitated in 10% (v/v) trichloroacetic acid. Protein pellets were solubilized in 50 μl of Novex LDS Sample Buffer(Thermo Fisher) containing 0.1 M Tris Base and NuPage Sample reducing agent (Thermo Fisher). Precipitated proteins were run on SDS-PAGE gels and were transferred onto nitrocellulose membranes. Immunodetection assays were performed according to standard procedures. NHBA full-length protein and its C-terminal fragments were identified with polyclonal mouse antisera raised against the recombinant NHBA full-length protein (working dilution 1:1000) or against the recombinant C2 fragment (working dilution 1:1000) [15], produced in-house. hLf was detected with immunodetection analysis using anti-Lactoferrin rabbit Polyclonal Antibody (PA5-29744 ThermoFisher Scientific) (working dilution 1:500). An anti-mouse antiserum (for NHBA) and anti-rabbit antiserum conjugated to horseradish peroxidase (32230 and 32260 Thermo Fisher Scientific) was used as secondary antibody. Bands were visualized with Super Signal West Pico Chemiluminescent Substrate (Thermo Fisher Scientific) following the manufacturer’s instructions.

### Expression and purification of recombinant NHBA proteins

Recombinant wild type and Δ-Arg NHBA protein was expressed in *E*. *coli* BL21 (DE3) strain by using EnPresso B growth kit (BioSilta) supplemented with 100 μg/ml ampicillin. Bacteria were grown at 30°C for 16 h, and protein expression was induced by the addition of 1 mM isopropyl β-D-1-thiogalactopyranoside (IPTG) (Sigma) at 25°C for 24 h. The soluble proteins were extracted by sonication in 50 mM NaH_2_PO_4_, 300 mM NaCl, 10 mM imidazole, pH 8.0, supplemented with protease inhibitors (cOmplete, EDTA-free Protease Inhibitor Cocktail, Roche), followed by centrifugation to remove cell debris. Recombinant proteins were purified from 0.22 μm cut-off filtered supernatant by affinity chromatography with HisTrap HP column (GE Healthcare) using an AktaPurifier System (GE Healthcare) following the recommended procedure. After washing, elution was performed applying a gradient from 0 to 250 mM imidazole in 20 column volumes (CVs). Eluted sample was dialyzed at 4°C overnight against 10 mM NaH_2_PO_4_, pH 7.0, buffer using Snake Skin Dialysis Tubing, 10,000 MWCO, 22 mm (Thermo Scientific), and a second step of purification was performed to obtain a highly pure product. The dialyzed wild tipe NHBA sample was loaded onto a HiTrap heparin HP column (GE Healthcare) using an AktaPurifier System (GE Healthcare). After washing, elution was performed by applying a gradient from 0 to 500 mM NaCl. The dialyzed Δ-arg NHBA sample was loaded onto a HiTrap Q HP column (GE Healthcare) using an AktaPurifier System (GE Healthcare), followed by a gradient from 0 to 500 mM NaCl. For both samples, to exchange the buffer, eluted samples were dialyzed at 4 °C overnight against phosphate buffered saline (PBS) using Snake Skin Dialysis Tubing, 10,000 MWCO, 22 mm (Thermo Fisher Scientific). Protein content was quantified using the BCA Kit (Thermo Fischer Scientific). Purity was checked by SDS-PAGE analysis, as well as by SEC-UPLC loading the sample on BEH200 4.6x300mm column (Waters). Size exclusion chromatography was performed using 10 mM NaH_2_PO_4_, 400 mM (NH_4_)_2_SO_4_, pH 6.0, buffer. Endotoxin content was quantified by LAL assay using ENDOSAFE instrument (Charles River).

### NHBA cleavage *in vitro* by saliva and human kallikrein1

Proteins were used starting from a stock concentration of 17 μM for NHBA recombinant protein (obtained as previously described), 17 μM for recombinant hK1 (2337-SE R&D System) and 3.4 μM for PKa purified from human plasma (K2638 Sigma Aldrich). Both Kallikreins were diluted to 0.17 μM and incubated at different dilutions with NHBA: from 1:5 (v/v) (final molar ratio 1:500) to 1:5,000(v/v) (final molar ratio 1:500,000) between hK1, PKa and NHBA. NHBA was incubated with saliva (Human saliva lot BRH1325364 from Seralab) in a ratio of 1 ml of saliva per mg of recombinant proteins. For inhibition assay Phenylmethanesulfonyl fluoride (PMSF, P7626 from Sigma-Aldrich) was first incubated 10 minutes at room temperature with hK1 at final concentration 1 mM and 6 mM, followed by addition of recombinant NHBA. Samples were incubated at 37°C overnight and the reaction was stopped by the addition of loading sample buffer for SDS-PAGE; then samples were centrifuged for 1 minute and analysed on SDS-PAGE.

### Proteomic analysis of saliva

For proteomic analysis, human saliva (Human saliva lot BRH1325364 from Seralab) was fractionated by anion exchange chromatography. Dialyzed saliva was loaded on a Mini Q PE 4.6/50 column (GE Healthcare; Ȓ 0.8 ml CV), with a flow of 0.2 ml/min on an Acquity-HPLC system (Waters) previously equilibrated with buffer 25 mM Tris pH 8.0.

Elution was performed with a flow of 1 ml/min in a gradient mode from 25 mM Tris, pH 8.0 to 25 mM Tris HCl 0.5 M NaCl, pH 8.0 in 20 CV and proteins were collected in fractions of 0.5 ml volume. NHBA cleavage activity of each fraction was evaluated by incubating NHBA at a concentration of 1 mg/ml with the same volume of each fraction as previously described. Protein fractions positive for proteolytic activity on NHBA protein were pooled and precipitated in 10% (v/v) trichloroacetic acid and 0.04% (w/v) sodium deoxycholate. Protein pellets were solubilized in 50 μl of 0.1% (w/v) Rapigest (Waters, MA, USA) and 1mM DTT and 50 mM ammonium bicarbonate, boiled at 100°C for 10 min. After cooling down, 1μg of LysC/trypsin mix (Promega) was added and the reaction was performed overnight. Digestions were stopped with 0.1% final formic acid, desalted using OASIS HLB cartridges (Waters) as described by the manufacturer, dried in a Centrivap Concentrator (Labconco) and resuspended in 100 μl of acetonitrile (ACNcan) 3% (v/v) and formic acid 0.1% (v/v). An Acquity HPLC instrument (Waters) was coupled on-line to a Q Exactive Plus (Thermo Fisher Scientific) with an electrospray ion source (Thermo Fisher Scientific). The peptide mixture (10 μl) was loaded onto a C18-reversed phase column Acquity UPLC peptide CSH C18 130Å, 1.7μm 1 x 150 mm and separated with a linear gradient of 28–85% buffer B (0.1% (v/v) formic acid in ACN) at a flow rate of 50 μL/min and 50 °C. MS data were acquired in positive mode using a data-dependent acquisition (DDA) dynamically choosing the five most abundant precursor ions from the survey scan (300–1600 *m/z*) at 70,000 resolution for HCD fragmentation. Automatic Gain Control (AGC) was set at 3E+6. For MS/MS acquisition, the isolation of precursors was performed with a 3 *m/z* window and MS/MS scans were acquired at a resolution of 17,500 at 200 *m/z* with normalized collision energy of 26 eV. The mass spectrometric raw data were analyzed with the PEAKS software ver. 8 (Bioinformatics Solutions Inc., ON, Canada) for *de novo* sequencing, database matching and identification. Peptide scoring for identification was based on a database search with an initial allowed mass deviation of the precursor ion of up to 15 ppm. The allowed fragment mass deviation was 0.05 Da. Protein identification from MS/MS spectra was performed against NCBInr *Homo sapiens* (Human) protein database (112,970,924 protein entries; 41,399,473,309 residues) combined with common contaminants (human keratins and autoproteolytic fragments of trypsin) with a FDR set at 0.1%. Enzyme specificity was set as C-terminal to Arg and Lys, with a maximum of four missed cleavages. N-terminal pyroGlu, Met oxidation and Gln/Asn deamidation were set as variable modifications.

### Purification of NHBA fragments after cleavage

NHBA recombinant protein was digested with saliva and recombinant hK1 as previously described and the obtained digested fragments were separated on His Spin Trap devices (GE Healthcare) following the manufacturer’s procedure. The His Spin Trap was equilibrated with 50 mM NaH_2_PO_4_, 300 mM NaCl, pH 8.0, NHBA cleaved recombinant protein was loaded on the devices and after washing C-fragments were eluted with 50 mM NaH_2_PO_4_, 300 mM NaCl, 500 mM Imidazole, pH 8.0.

### Mass Spectometry analysis of the cleaved purified C-term fragments

The C-terminal fragments recovered from His Spin Trap elution fraction were analysed by Mass Spectrometry. The acidified protein solutions were loaded onto a Protein Microtrap cartridge (from 60 to 100 pmols), desalted for 2 min with 0.1% (v/v) formic acid at a flow rate of 200 ml/min and eluted directly into the mass spectrometer using a step gradient of acetonitrile [55% (v/v) acetonitrile, 0.1% (v/v) formic acid]. Spectra were acquired in positive mode on a SynaptG2 HDMS mass spectrometer (Waters) equipped with a Z-spray ESI source. The quadrupole profile was optimized to ensure the best transmission of all ions generated during the ionization process. Mass spectra were smoothed, centroided and deconvoluted using MassLynx vers. 4.1 (Waters).

### Bacterial strains and growth conditions

*N*. *meningitidis* wild type (wt) strain MC58 [22] and the ΔNalP and ΔNHBA MC58 strains [12] were cultured on GC agar plates (Difco) at 37 °C plus 5% CO_2_. Overnight colonies grown on GC agar plate were used to start a culture in 25 ml of GC medium (Difco) in 125 ml flask. An OD_600nm_ ~ 0.1 was used as the start culture density. Liquid culture was grown at 32 °C plus 5% CO_2_, under aerobic condition (180 rpm) until OD_600nm_ ~ 0.4 and then used for experimentation. 2.5 ml of cultured bacteria were incubated with 0.2 ml of saliva (Human saliva lot BRH1325364 from Seralab), 15 μl of human plasma purified kallikrein (PKa) (K2638 Sigma Aldrich, 3.4 μM) or 0.2 ml of liquid GC medium, as negative control, for 30 minutes at 32°C. Culture medium supernatant was recovered and used for immunodetection analysis while bacteria were used for FACS analysis.

### Flow Cytometry analysis

Bacteria, obtained as previously described, were washed and incubated for 1 hour, at room temperature, with polyclonal mouse antiserum raised against an NHBA C-terminal fragment. After a washing step, samples were incubated for 30 minutes, at room temperature, with Alexa flour 488-conjugated goat anti-mouse IgG secondary antibody (Life Technologies). All washing steps and antibody dilutions were performed using 1% (w/v) BSA in PBS. Labeled bacteria were washed and fixed for 1 hour, at room temperature, using 2% (v/v) formaldehyde (Carlo Erba Reagents) in PBS. Samples were analyzed with BD FACS CantoII system (BD Bioscience) using FlowJo software. Each condition was performed in triplicate.

## Results

### NHBA is cleaved by human saliva

**Fig 1:**
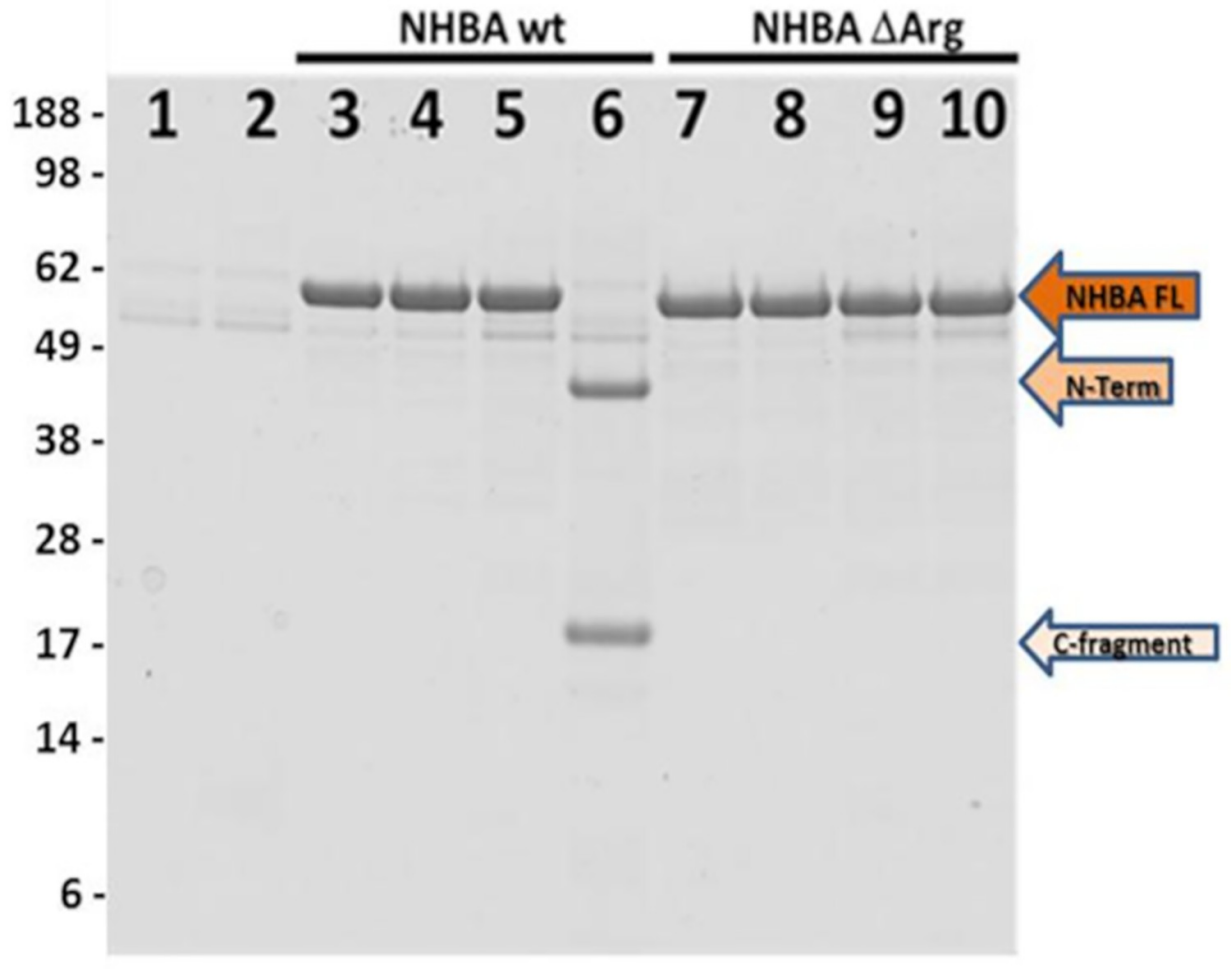
Saliva promotes NHBA cleavage. SDS PAGE of NHBA incubated with human saliva in PBS 1X. Recombinant proteins have been tested at 3.8 μM as final concentration and 1 ml of saliva per mg of protein was used. Lane 1: pool of saliva; lane 2: pool of saliva incubated for 4 hours at 37°C; lane 3: NHBA wild type recombinant protein; lane 4: NHBA wild type recombinant protein incubated for 4 hours at 37°C; lane 5: NHBA wild type recombinant protein and saliva mixed together without incubation; lane 6: NHBA wild type recombinant protein incubated with saliva 4 hours at 37°C; lane 7: NHBA ΔArg recombinant protein; lane 8: NHBA ΔArg recombinant protein incubated for 4 hours at 37°C; lane 9: NHBA ΔArg recombinant protein with saliva mixed together without incubation; lane 10: NHBA ΔArg recombinant protein incubated with saliva for 4 hours at 37°C.

Lactoferrin is known to be present in various biological fluids, such as blood, milk, tears, nasal secretions and saliva, and it has been reported to be a protease responsible for NHBA cleavage [12]. To explore the possibility that NHBA protein may be processed during initial *Neisseria* colonization of the nasopharynx, we assessed whether saliva, which contains hLF, was able to proteolytically cleave the protein. To this end, human saliva from a pool of donors was incubated with recombinant NHBA (from NZ98/254 strain) protein overnight at 37°C. Samples were recovered and analyzed by SDS-PAGE. As shown in Fig 1, saliva treatment of NHBA protein gave rise to two main fragments with an apparent molecular weight of approximately 40 kDa and 20 kDa (lane 6), similarly to those produced by hLF [12]. Significantly, a recombinant NHBA mutant protein, wherein all Arg residues of the Arg-rich region had been deleted (NHBA ΔArg), was not cleaved by saliva (lanes 9 and 10), indicating that the presence of the Arg-rich region was necessary for the proteolytic cleavage.

### Lactoferrin is not the main actor mediating NHBA cleavage in human saliva

**Fig 2:**
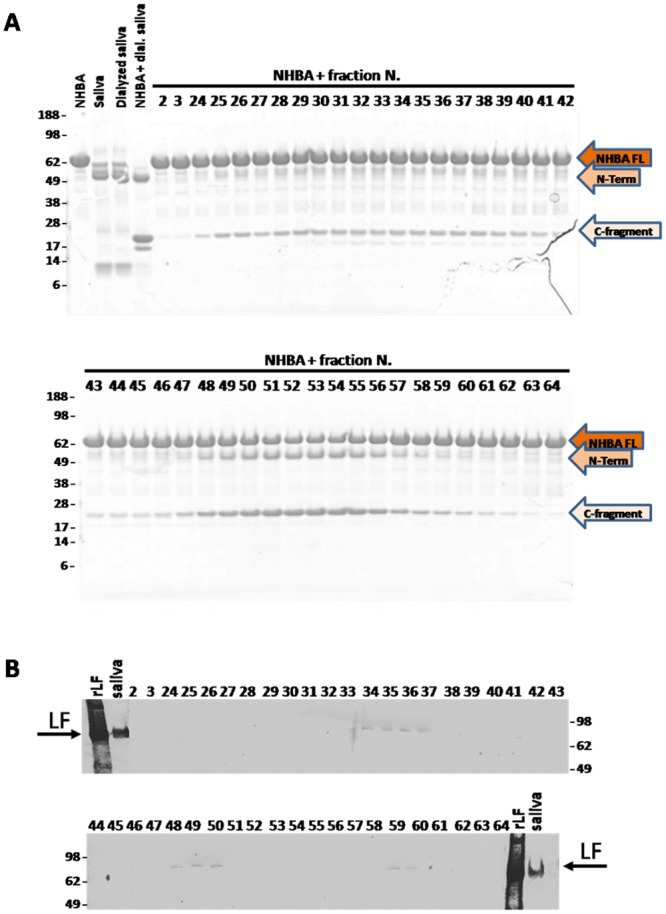
Lactoferrin in Saliva fractions and their activity on NHBA cleavage. A) SDS PAGE of the fractions obtained through the saliva partial purification incubated with NHBA. All the samples were maintained at 37°C overnight and then loaded on SDS-PAGE. In the first four lanes, NHBA recombinant protein, saliva, saliva post dialysis and saliva post dialysis incubated with NHBA as reported as control. From number 2 to number 64, are reported the different fractions obtained from saliva purification after incubation with NHBA recombinant protein. NHBA at a concentration of 1 mg/ml was incubated with the same volume of each fraction. B) Immunodetection on saliva recovered fractions against Lactoferrin. Recombinant Lactoferrin (rLF), saliva (saliva) and the fractions from AEC were loaded on the gel. Lactoferrin is present in frations from 34–37, 48–50 and 59–60.

**Fig 3:**
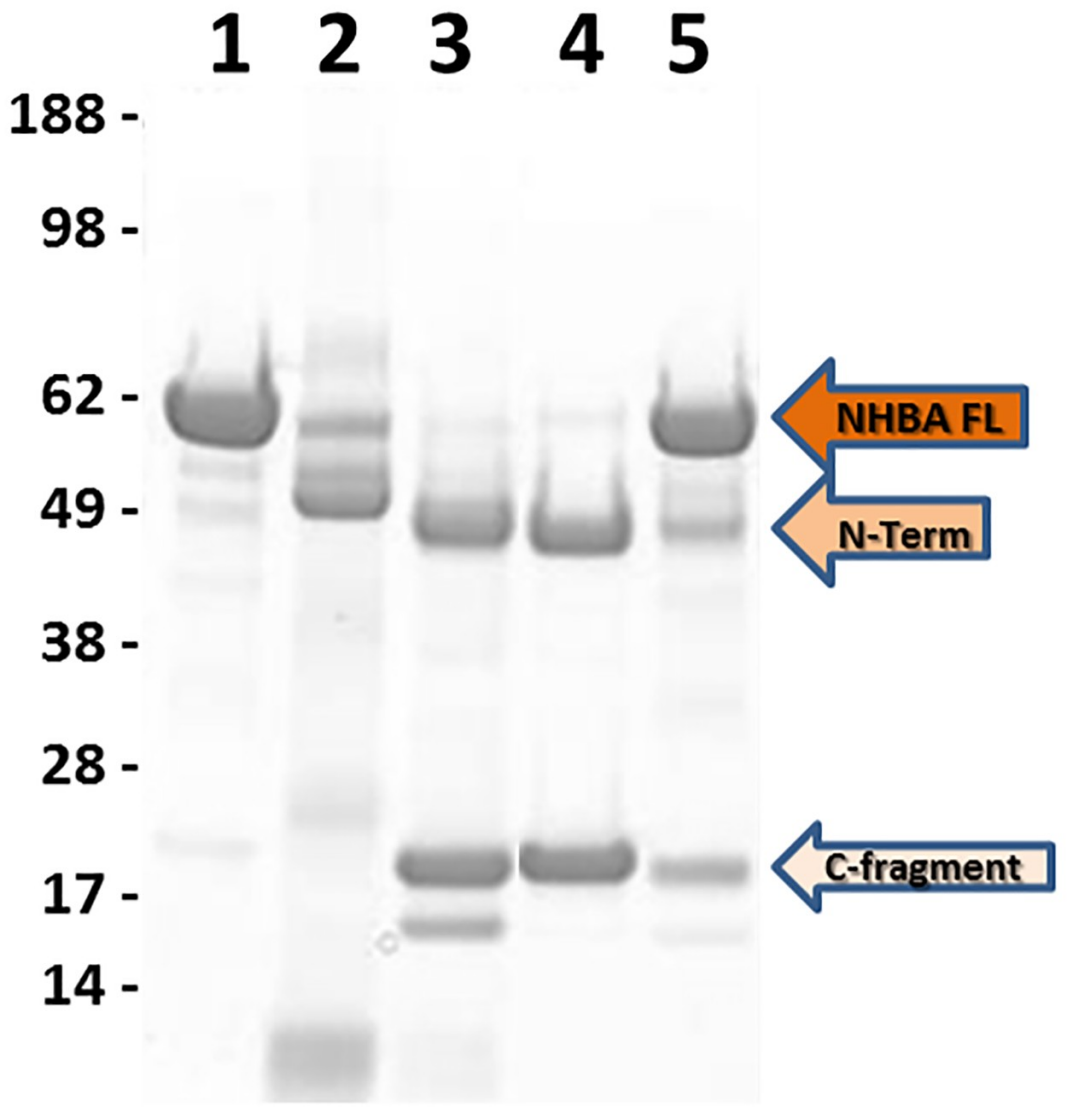
Lactoferrin in saliva is not the main factor responsible for NHBA cleavage. SDS-PAGE on NHBA processed by recovered and concentrated saliva fractions. All the samples were maintained at 37°C overnight and then analyzed by SDS-PAGE. Lane 1: NHBA recombinant protein; lane 2: saliva; lane 3: NHBA recombinant protein incubated with saliva; lane 4: NHBA recombinant protein incubated with concentrated pool of fractions from 52–54; lane 5: NHBA recombinant protein incubated with concentrated pool of fractions positive for lactoferrin in immunodetection.

To verify whether the protease responsible for NHBA cleavage in saliva was the hLF, proteins contained in saliva were partially fractionated by Anion Exchange Chromatography. Proteins were eluted by increasing ionic strength (S1 Fig), and each recovered fraction was examined for both the ability to cleave NHBA protein and for the presence of hLf. The proteolytic activity of each fraction on NHBA protein was monitored by SDS-PAGE analysis (Fig 2A), while the presence of hLf was assessed by immunodetection-analysis (Fig 2B), using Lactoferrin Polyclonal Antibodies (commercially available). Surprisingly, while the hLF was mainly detected in fractions from 33 to 37, from 48 to 50 and 59 and 60, the majority of the proteolytic cleavage of NHBA was observed from fraction 50 to fraction 56, suggesting that hLF was not the main factor, in saliva, responsible for cleavage of NHBA. To further confirm this experimental evidence, either the fractions with major cleavage activity (52–54) or those containing hLF (33–37) were pooled and concentrated, and their ability to cleave NHBA was tested. As shown in Fig 3, a pool of fraction 52–54 completely processed NHBA protein after overnight incubation at 37°C (lane 4). On the contrary, only a minimal activity was visible for a pool of fractions 33–37 positive for hLf (lane 5). These experimental evidences led to the conclusion that, in addition to hLF, another unknown human protease in saliva was able to cleave NHBA protein and the cleavage of NHBA in saliva resulted mainly from its activity.

### Kallikrein 1 is the main factor responsible for NHBA cleavage in human saliva

**Fig 4:**
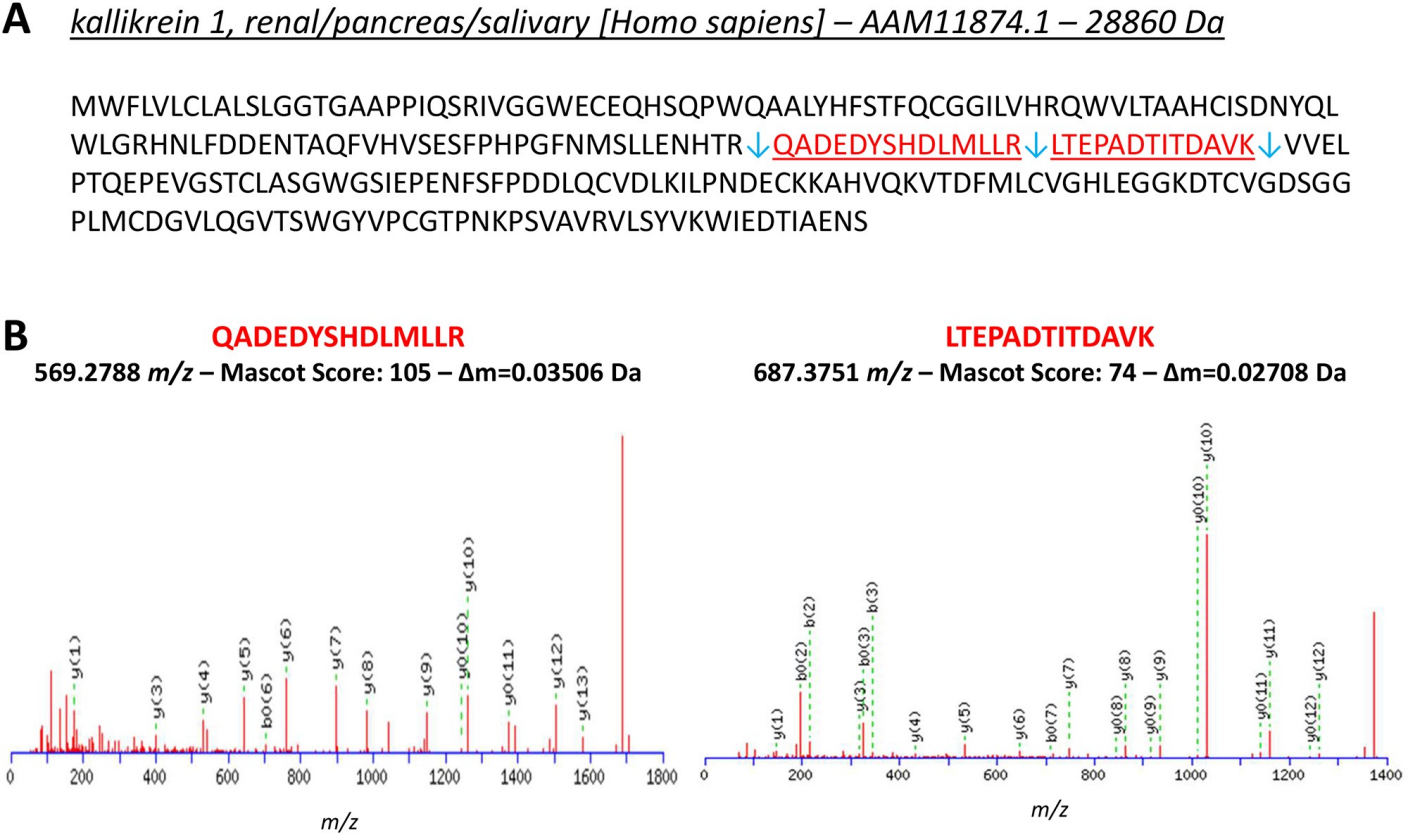
The protease kallikrein 1 was identified in NHBA-cleaving fractions from human saliva. **(A)** Primary sequence of hK1 identified from saliva fractions able to cleave NHBA. Identified peptides by LC-MS/MS are highlighted in red. Blue arrows indicate trypsin cleavage sites. **(B)** MS/MS spectra of the peptides identified from hK1: m/z values, Mascot score and experimental delta mass are reported.

**Fig 5:**
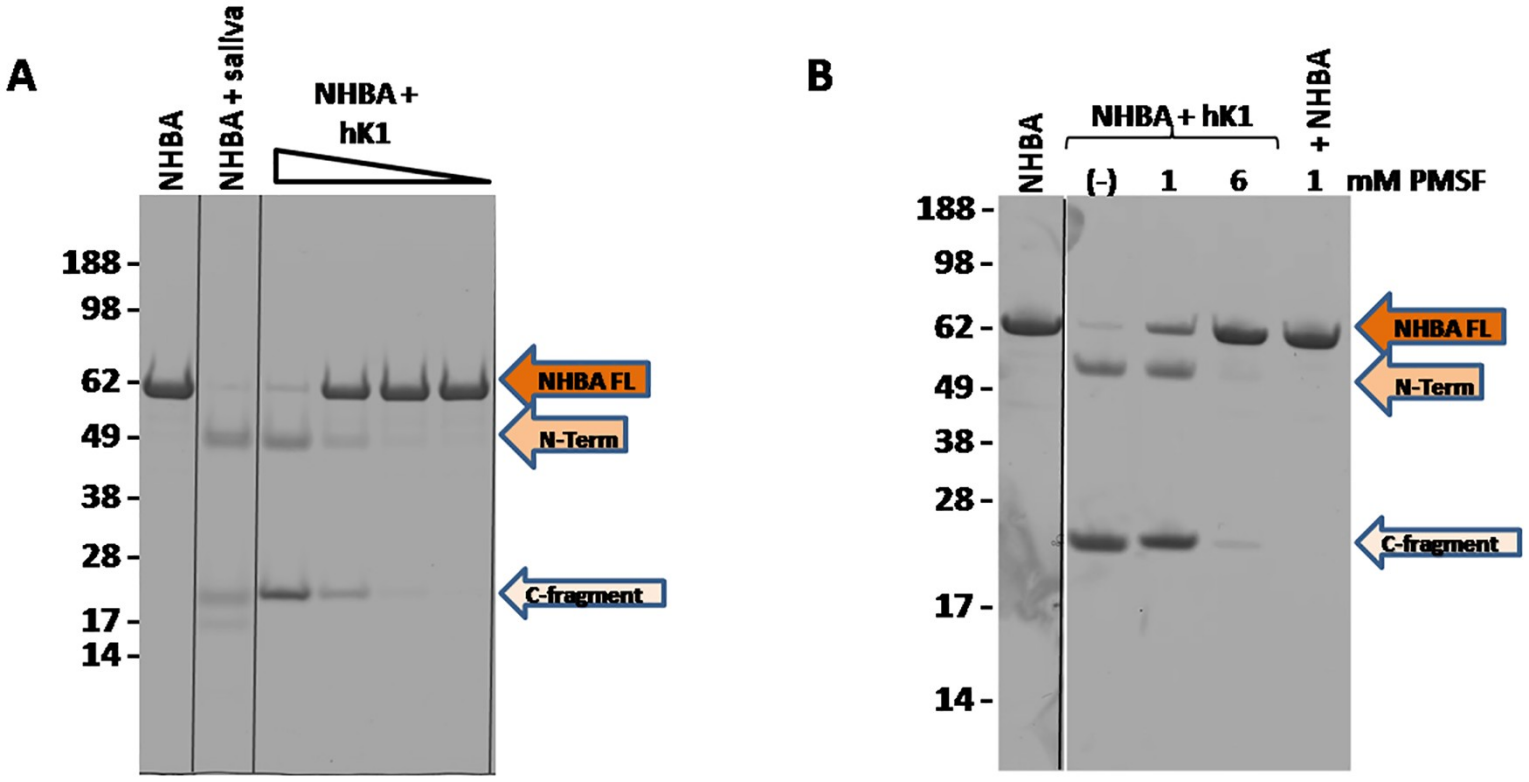
Kallikrein (hK1) processes NHBA *in vitro*. A) SDS-PAGE of NHBA cleavage by hK1. All the samples were maintained at 37°C overnight and then loaded on SDS-PAGE. Lane 1: NHBA; Lane 2: NHBA incubated with human saliva (as previously described); Lane 3–6: NHBA incubated with hK1 at a molar ratio ranging from 1:500, 1:5,000, 1:50,000 to 1:500,000 between hK1 and NHBA. B) Lane 1: NHBA; Lane 2: NHBA incubated with recombinant hK1 (1:500 molar ratio between hK1 and NHBA); lane 3–4: NHBA incubated with recombinant hK1 (1:500 molar ratio between hK1 and NHBA) and its specific inhibitor Phenylmethanesulfonyl fluoride (PMSF) at 1 mM and 6 mM concentration respectively; Lane 5: NHBA incubated with 1 mM Phenylmethanesulfonyl fluoride (PMSF).

To identify the main protease responsible for NHBA processing in saliva, a pool of fractions (52–54) with the highest NHBA proteolytic activity was analyzed by mass spectometry based proteomics. Proteins were precipitated, digested with trypsin and the resulting peptides were subjected to LC-MS/MS analysis. Protein identification from mass spectrometry data was performed against the *Homo sapiens* public database available at NCBInr. Among the identified proteins, we found the protease kallikrein 1 (hK1), whose identity was assigned by the MS/MS sequence of two peptides (Fig 4). This isoform of kallikrein is specifically expressed in human kidney, pancreas and salivary glands and its cleavage site is located downstream a positively charged amino acid (such as Lys or Arg residues) [21]. Hence, hK1 was considered a plausible candidate for cleavage of NHBA in saliva, since NHBA contains an Arg-rich region that is highly conserved among different *N*. *meningitidis* strains and that could act as target for hK1 proteolytic cleavage. To further investigate this matter, recombinant hK1 and human saliva were incubated overnight at 37°C with recombinant NHBA protein. SDS-PAGE analysis showed that recombinant hK1 (lane 3–6, Fig 5A) was able to proteolytically cleave NHBA with fragmentation patterns identical to that induced in saliva (lane 2, Fig 5A). Proteolytic cleavage resulted in the generation of two main fragments (highlighted with arrows in Fig 5A), whose amount is directly linked to the quantity of kallikrein, in a dose-dependent manner, with molar ratio ranging from 1:500 to 1:500,000 between hK1 and NHBA. Moreover, the NHBA fragments generated by the cleavage had an apparent molecular weight comparable to the fragments produced by hLf cleavage [12]. To further confirm the hK1 specific cleavage of NHBA, the same reaction was conducted in presence of Phenylmethanesulfonyl fluoride (PMSF), a specific hK1 inhibitor reported previously [23]. As shown in Fig 5B, incubation of hK1 with PMSF at two different concentrations, reduced (lane 3) and blocked (lane 4) the cleavage of NHBA, while hK1 cleaved NHBA (lane 2) and PMSF alone had no effect on cleavage (lane 5). Moreover, human plasma kallikrein (PKa), was also tested for its capability to process NHBA. PKa hydrolyzed NHBA in the similar pattern as hK1 (S2 Fig), but it is more effective, as expected, since it is purified from Human plasma and it is not recombinant as the used hK1. These results indicated that hK1 is the main protease responsible for NHBA processing in the saliva and suggest that NHBA could be a substrate also for plasma kallicrein.

### Kallikrein and human lactoferrin promote the formation of two identical fragments

To identify the exact cleavage site within the NHBA protein promoted by hK1 and saliva, we purified the C-terminal fragments generated by their cleavage. Therefore, NHBA was incubated with saliva or with hK1 overnight at 37°C and the resulting fragments were separated by affinity chromatography. Since NHBA was His-tagged at the C-terminus, we used Ni-NTA resin to bind and purify the cleaved C-terminal fragments, while N-terminal fragments were not bound to the column and were released in the flow through (Fig 3S).

**Fig 6:**
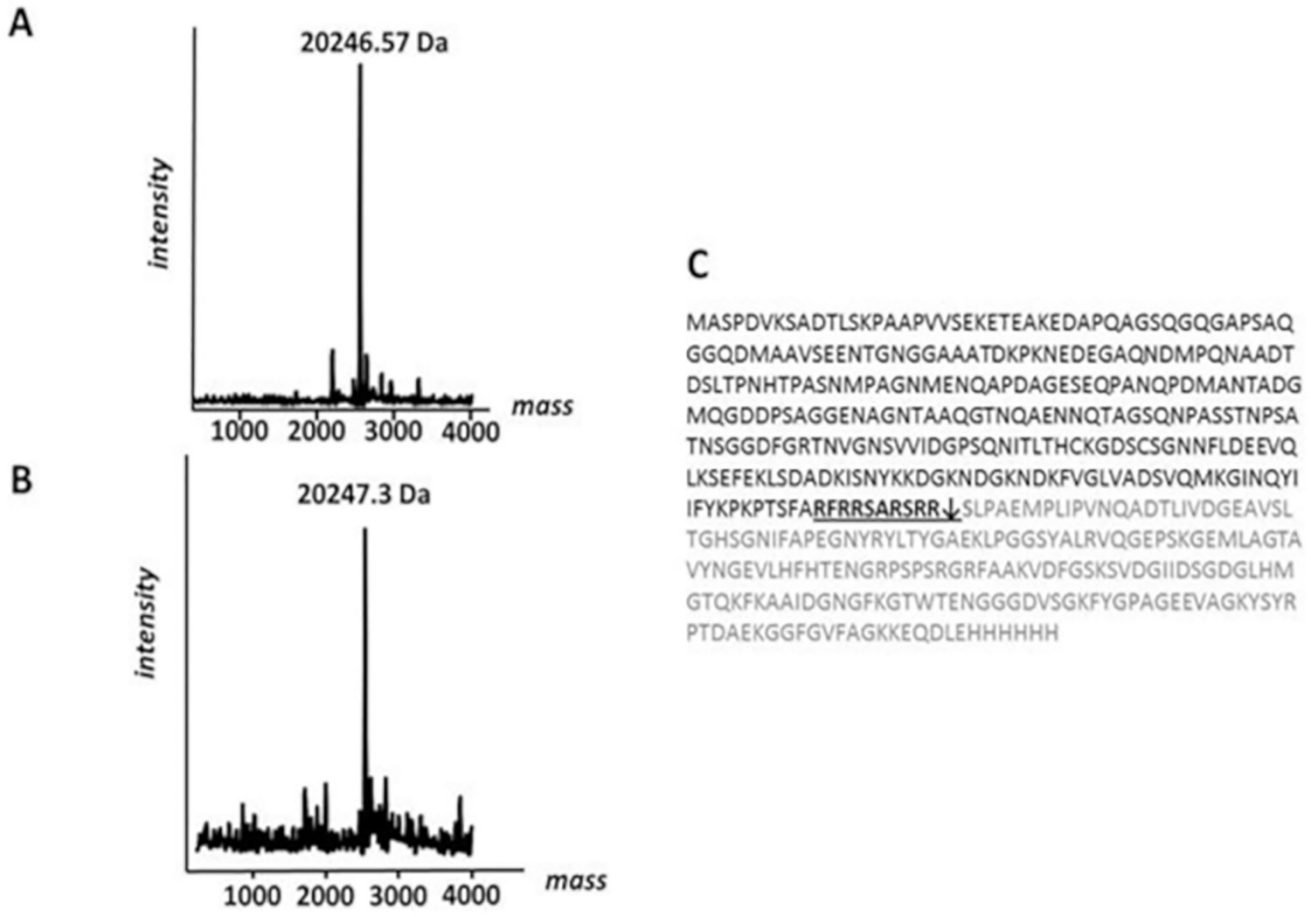
Intact mass measurement of NHBA C-terminal fragment obtained from saliva or kallikrein cleavage. Intact mass determination was performed by ESI-Q-TOF. A) The NHBA C-fragment after saliva cleavage showed an observed molecular weight of 20,246.57 Da, compatible with the primary sequence of C-terminal region of NHBA starting from Ser^288^. B) The NHBA fragment after cleavage by recombinant hK1 showed a molecular weight of 20,247.3 Da. C) Amino acid sequence of recombinant NHBA (NZ) full-length protein (UniProtKB code A0A0E0TT97): Arg-rich motif is indicated in bold, cleavage site of epithelial cells proteases is indicated with a black arrow and amino acid sequence of newly generated C-terminal fragment is highlighted in gray.

Once the cleaved C-term fragments generated by human saliva and hK1 were purified, they were analyzed by mass spectrometry to determine their precise molecular weight, thus allowing identification of the cleavage site of hK1 and saliva within the NHBA protein. Intact mass determination was performed by positive electrospray ionization using a Q-TOF mass spectrometer. For the NHBA C-fragment derived from saliva cleavage, we detected a molecular weight of 20,246.57 ± 0.14 Da that was compatible with the primary sequence of the C-terminal region of NHBA starting from Ser^288^ (Fig 6A). Similarly, the NHBA fragment derived from recombinant hK1 cleavage showed a molecular weight of 20,247.3 Da ± 2.0 Da (Fig 6B) that was also compatible with the same C-terminal sequence starting from Ser^288^. We therefore concluded that the cleavage site of both hK1 and saliva was located immediately downstream of the Arg-rich region at the level of Ser^288^, and corresponded to the previously reported cleavage site of hLF [12].

### NHBA and kallikrein in *Neisseria meningitidis*

Having demonstrated that *in vitro* hK1 was the main protease of saliva responsible for the proteolytic cleavage of NHBA into the N- and C-terminal fragments and that also PKa, present in the plasma is able to process NHBA *in vitro* with the same pattern (supplementary Fig 2), we further verified the ability of both saliva and PKa to process NHBA when expressed on the surface of live bacteria.

**Fig 7:**
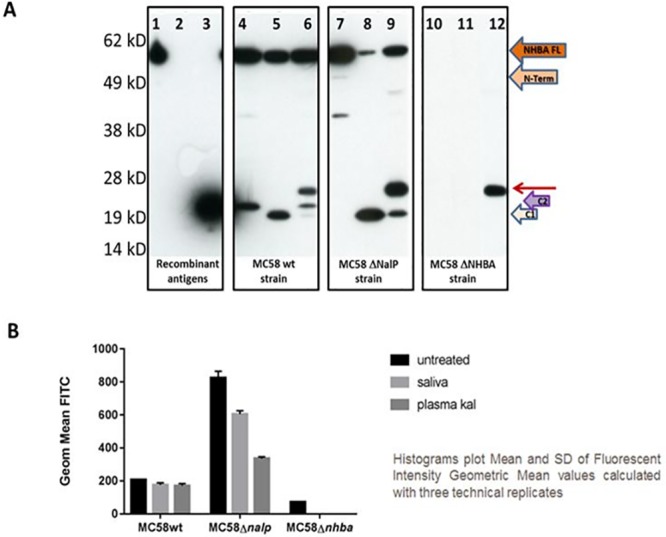
NHBA cleavage by human plasma kallikrein (PKa) and saliva occurs on live bacteria. Immunodetection, using α-NHBA-C-fragment polyclonal antibody. Lane 1: NHBA wild type recombinant protein; lane 2: NHBA-AB recombinant fragment; lane 3: NHBA recombinant C-fragment; lane 4: MC58 wild type supernatant; lane 5: MC58 wild type supernatant following incubation with PKa; lane 6: MC58 wild type supernatant following incubation with saliva (containing hK1); lane 7: MC58 ΔNalP supernatant; lane 8: MC58 ΔNalP supernatant following incubation with PKa; lane 9: MC58 ΔNalP supernatant following incubation with saliva; lane 10: MC58 ΔNHBA supernatant; lane 11: ΔNHBA supernatant incubated with PKa; lane 12: MC58 ΔNalP supernatant incubated with saliva (containing hK1). B) FACS analysis of MC58wt, MC58Δnalp and MC58Δnhba strains stained with anti-NHBA-C-terminal-FITC antibody. Bacteria were incubated for 30 minutes with PKa, saliva or with medium, as negative control (3 technical replicates). Histograms plot the geometric mean of Fluorescence Intensity (FITC). Error bars denote standard deviation.

To this aim, *Neisseria meningitidis* MC58 wild-type strain and the relative knock-outs for *nalP* (gene coding for the meningococcal serine protease able to cleave NHBA) and *nhba* genes, as a control strains, were grown at 32°C to simulate the nasopharyngeal environment [24], and then treated with saliva or PKa. After 30 minutes, samples were collected and the presence of the NHBA C-terminal domain was assessed either into supernatants by immunodetection analysis (Fig 7A) or on bacterial surface by FACS analysis (Fig 7B), using mouse polyclonal antibodies against C2 fragment produced in house. As previously reported [12], the NHBA C-terminal fragment derived from the cleavage of meningococcal NalP protease, named C2, could be detected in the growth supernatant of MC58 wt (Fig 7A, lane 4). In addition, a shorter NHBA C-terminal fragment was observed in the supernatant of bacteria incubated with PKa (Fig 7A, lane 5) with an apparent molecular weight identical to the NHBA C-terminal fragment generated by hLF, named C1 [12]. When the bacteria were incubated with saliva, NHBA was processed mainly in the C2 fragment (lane 6) and only a small amount of C1 was observed, probably generated by NalP cleavage. In the case of MC58Δ*nalp* strain, the NHBA C-terminal fragment was only detected in the supernatant of bacteria incubated with PKa or saliva (Fig 7A, lane 8 and 9), confirming hat their proteolytic activity led to an accumulation of NHBA C-terminal fragment into the supernatant. Of note, the NHBA full-length protein was only detected in MC58 wt (lanes 4–6) and MC58Δ*nalp* strain (lanes 7–9), but not in the MC58 Δ*NHBA* strain (lanes 10–12). The band detected at higher molecular weight than the C-fragment (red arrow in lane 6, 9 and 12), present in all lanes of the samples treated with saliva, results from an unspecific reaction of the polyclonal serumwith saliva.

These data show that NHBA C-fragments generated by incubation with either, PKa or hK1 present in saliva, have identical molecular weights, a bit lower than the molecular weight of the C2fragment generated by NalP cleavage.

Evidence that PKa and hK1 cleaved NHBA protein on live bacteria was further supported by FACS analysis. Therefore, following protease treatments, bacteria were stained with anti-NHBA-C-terminal–FITC antibody and the amount of the uncleaved C-terminal domain measured by fluorescent intensity. As reported in Fig 7B, no differences were observed in the amount of C-terminal domain detected on the surface of MC58wt treated with PKa, saliva or growth medium. The low amount of the NHBA C-terminal domain detected on the bacterial surface is due to the NalP proteolytic cleavage. On the contrary, in the case of MC58Δ*nalp* strain, in which the NHBA is not proteolytically cleaved by NalP, the NHBA is present on the bacterial surface in its full-length form, and the amount of NHBA C-terminal domain decreases only after treatment with PKa or saliva.

These results indicate that both hK1 present in saliva and PKa were able to cleave NHBA on live bacteria and generation and release of NHBA C-terminal domain from the bacterial surface resulted in the accumulation of C-terminal fragment int the supernatant.

### Discussion

The role of NHBA in *Neisseria meningitidis* pathogenesis is still unclear. Previous studies have shown that NHBA is able to bind Heparin and glycosaminoglycans (GAGs) on epithelial cells through the Arg-rich region [25] [13], supporting the hypothesis that NHBA can not only enhance bacterial survival in human serum, but may also play a crucial role during the adhesion step in *Neisseria meninigitidis* host colonization [15]. NHBA is cleaved by two specific proteases, NalP, a surface-exposed *Neisseria* serine protease, and hLf, present in several human biological fluids like serum, milk and saliva [12]. The C-terminal fragment of NHBA generated by proteolytic cleavage can induce a toxic effect on endothelial cells by altering the endothelial permeability [15]. In this study, we have assessed whether the proteolytic cleavage of NHBA could happen in saliva, since the nasopharynx is the only known *N*. *meningitidis* bacterial reservoir, and the first tissue where bacteria start colonization [24]. Understanding the mechanism which allows bacteria to pass through the human nasopharyngeal epithelium to start the invasive phase is a crucial step to develop a preventive treatment for meningitis disease [26]. For this purpose, we evaluated the possibility that NHBA could be processed by human saliva, the main fluid present in the nasopharyngeal tract. In this work, we demonstrate that NHBA is cleaved *in vitro* by human saliva into two main fragments, an N-terminal fragment (AB) and a C-terminal fragment (C1), and that C1 is the same fragment generated by hLf processing of NHBA; we found, however, that the main actor for NHBA processing in saliva is not hLf but hK1. The hK1 is a human serine protease that cleaves one amino acid downstream of positively charged amino-acids and is present in several biological fluids such as lymph, urine, saliva and pancreatic juice. We demonstrated that the hK1 cleavage site on NHBA happens on residue Ser^288^ just downstream of the Arg-rich region, confirming that hK1 processes NHBA at the same identical site previously reported for hLf [12]. Interestingly, also plasma Kallicrein (PKa) was also able to cleave NHBA, although the two serine proteases have different substrates and different specificity [18]. The proteolytic cleavage by the plasma Kallicrein, suggest that NHBA processing may even occur during *Neisseria* acute infection in the bloodstream, highlighting the potential role that NHBA could play at different stages of meningococcal pathogenesis.

We have so far identified four proteases able to cleave NHBA *in vitro*, upstream or downstream of the Arg-rich region: the meningococcal NalP protease (upstream cleavage), that generates the C2 fragment containing the Arg-rich motif, hLf, C3-convertase and hK1 (downstream cleavage), three human proteases generating the C1 fragment, devoid of the Arg-rich domain. Both fragments are released from bacteria into the external milieu [15] [12] [27].

We then tested the ability of NHBA to act as substrate for proteolytic cleavage by human saliva and PKa, when presented on the bacterial surface, in its natural environment. We showed that, in the MC58 wild-type strain, NHBA was proteolyzed generating two fragments, C2 and C1, deriving from proteolytic cleavage by NalP and saliva and PKa, respectively. In the case of ΔNalP MC58 strain, only human saliva and PKa had proteolytic activity, causing the release in the supernatant of only the C2 fragment.

Hence, we speculated whether the two different C-term fragments could play a different role in NHBA mediated *Neisseria* pathogenesis.

The important role of the NHBA Arg-rich region was demonstrated previously for heparin binding [12] and for *Neisseria meningitidis* adhesion to human epithelial cells [13].

It was also shown that C2, but not C1, increases endothelial permeability with a mechanism involving the production of oxygen radicals and the phosphorylation and degradation of adherents-junction protein VE-cadherin. [15]. Since the C2 fragment contains the Arg-rich domain, it is plausible that this fragment might interact with secreted or cell-associated proteoglycans in order to exert its biological role [15]. Moreover, the C2 fragment seems to be critical for invasion of human tissues, hence for virulence, and different studies showed the possibility that it is naturally released in the bacterial environment. In the case of hLf cleavage of NHBA, inducing the release of the C1 fragment, devoid of the Arg-rich domain, it has been speculated that such proteolytic cleavage could impair nasopharyngeal colonization, adhesion to epithelial cells and invasion into the bloodstream [15]. Therefore, the proteolytic cleavage by hK1, could also play an important role in meningococcal pathogenesis. Moreover, the processing of NHBA by tissue and plasma Kallicrein suggests that the two proteases could influence virulence at the mucosal and systemic level, respectively.

The Arg-rich region is highly conserved among different NHBA proteins and across MenB strains [12], suggesting a crucial role in NHBA function. However, there is yet no concrete explanation why, in the pharyngeal tract and bloodstream, four proteases are involved in NHBA processing, with meningococcal NalP targeting the Arg-rich region upstream, and kallikreins, C3-convertase [27] and lactoferrin [12] targeting it downstream. Bacterial colonization in the nasopharyngeal tract is the prerequisite for the *N*. *meningitidis* invasive phase. Successful colonizers must attach the epithelial lining, grow on the mucosal surface, evade the host immune response, and penetrate into the bloodstream. However, the invasive behavior is not part of the normal meningococcal life cycle since, once the colonizers have entered the bloodstream or the central nervous system, they cannot be easily transmitted to other hosts, and blood not only constitutes an immunologically challenging compartment but also an oxygen-limiting environment [28]. Environmental conditions which force the switch of *N*. *meningitidis* from carriage to invasive modes are not yet known, and the balance between the two kind of proteolytic cleavages, by NalP, producing C2 fragment or by hLf, C3-convertase or hK1 producing C1 fragment, may play a role in signaling to the bacteria the environmental changes required for initiation of the invasive phase. This aspect opens the possibility that NHBA processing by host proteases would not be solely a host defense mechanism, but could be crucial for the bacterium decision-making process in the carriage-to-virulence switch.

Further studies may consider investigating the environmental conditions which activate or inhibit NalP or kallikrein functions, and the influence that these cleavages could have on meningococcus virulence and invasiveness.

## Supporting information

S1 Fig Saliva fractionation on AEC. Chromatogram of the Anion Exchange chromatography. Red box highlights fractions with major protease activity on NHBA.
(TIF)

S2 Fig Tissue kallikrein (hK1) and plasma kallikrein (PKa) processes NHBA *in vitro*. SDS-PAGE of NHBA cleavage by human kallikrein. All the samples were maintained at 37°C overnight and then loaded on SDS-PAGE. Lane 1: NHBA alone; Lane 2: NHBA incubated with human saliva; Lane 3–6: NHBA incubated with recombinant tissue kallikrein 1 (hK1) at dilution from 1:500, 1:5,000, 1:50,000 and 1:500,000 molar ratio between NHBA and hK1; Lane 7–10: NHBA incubated with purified human plasma kallikrein (PKa) at dilution from 1:500, 1:5,000, 1:50,000 and 1:500,000 molar ratio between PKa and NHBA.
(TIFF)

S3 Fig NHBA fragments purification after digestion with saliva and hK1. A) SDS-PAGE of C-fragment purification after NHBA processing by saliva. Lane 1: NHBA recombinant protein alone; lane 2: NHBA recombinant protein incubated with saliva overnight at 37°C; lane 3: NiNtA flow thorught fractions; lane 4 and 5: NiNtA column washes; lane 6 and 7: elution fractions from the NiNtA column which contain the C-fragment. B) SDS-PAGE of C-fragment purification from NHBA processing by hK1, in a molar ratio 1:500 between hK1 and NHBA. Lane 1: NHBA recombinant protein; lane 2: NHBA recombinant protein incubated with hK1 overnight at 37°C; lane 3: NiNtA flow thorught fractions; lane 4 and 5: NiNtA column washes; lane 6 and 7: elution fractions from the NiNtA column which contain the C-fragment.
(TIF)
