# Supplementary figures and images for: NHBA is processed by kallikrein from human saliva
Source: PLoS One. 2019 Aug 1;14(8):e0203234. doi: 10.1371/journal.pone.0203234 (PMC6675046; doi:10.1371/journal.pone.0203234)

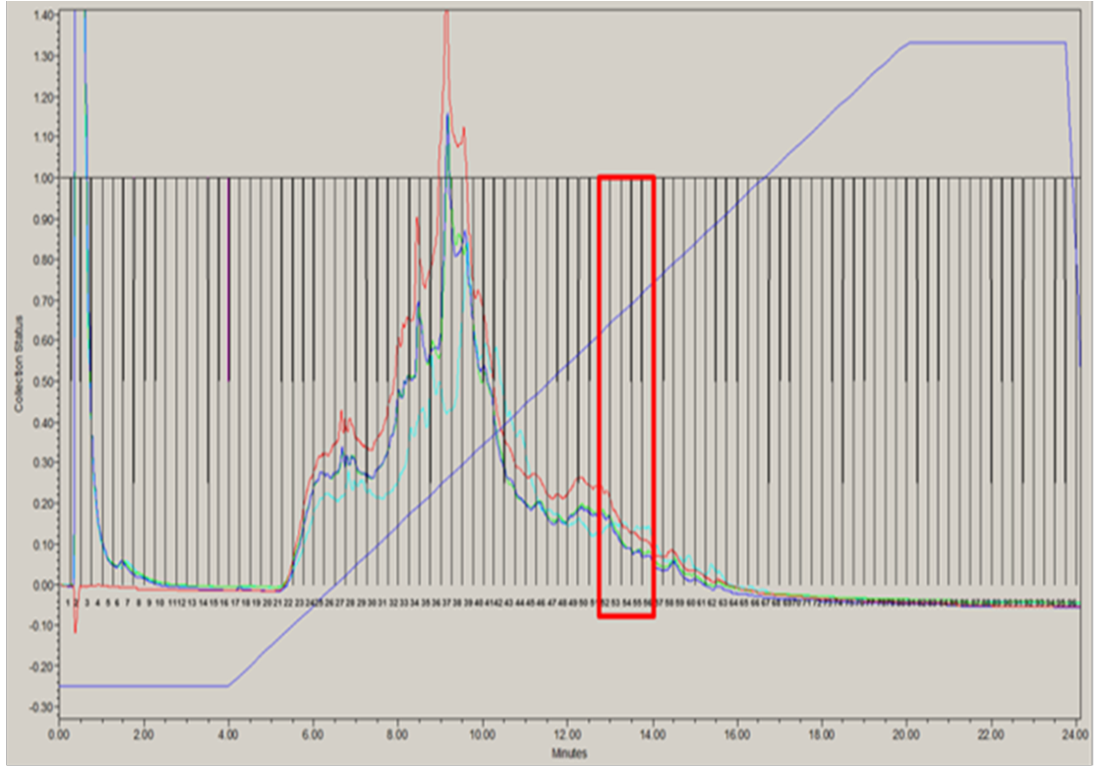

Supplement: S1 Fig — Chromatogram of the Anion Exchange chromatography. Red box highlights fractions with major protease activity on NHBA. (TIF) [file pone.0203234.s001.tif]

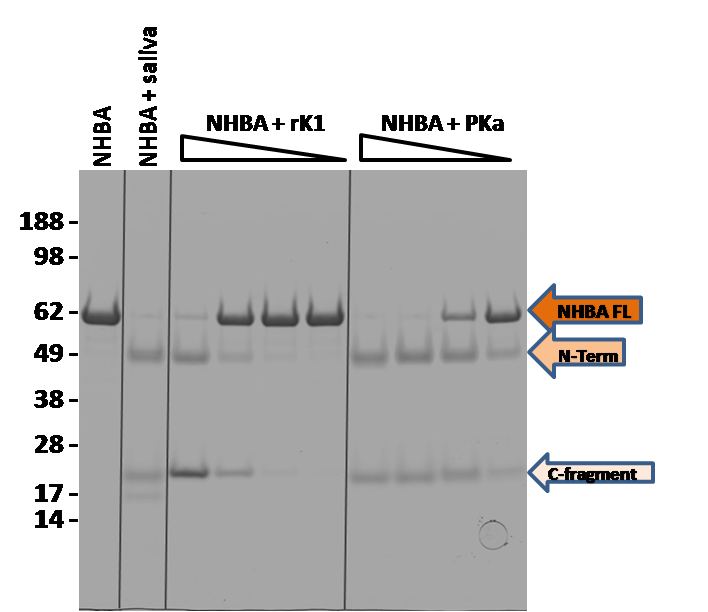

Supplement: S2 Fig — SDS-PAGE of NHBA cleavage by human kallikrein. All the samples were maintained at 37°C overnight and then loaded on SDS-PAGE. Lane 1: NHBA alone; Lane 2: NHBA incubated with human saliva; Lane 3–6: NHBA incubated with recombinant tissue kallikrein 1 (hK1) at dilution from 1:500, 1:5,000, 1:50,000 and 1:500,000 molar ratio between NHBA and hK1; Lane 7–10: NHBA incubated with purified human plasma kallikrein (PKa) at dilution from 1:500, 1:5,000, 1:50,000 and 1:500,000 molar ratio between PKa and NHBA. (TIFF) [file pone.0203234.s002.tiff]

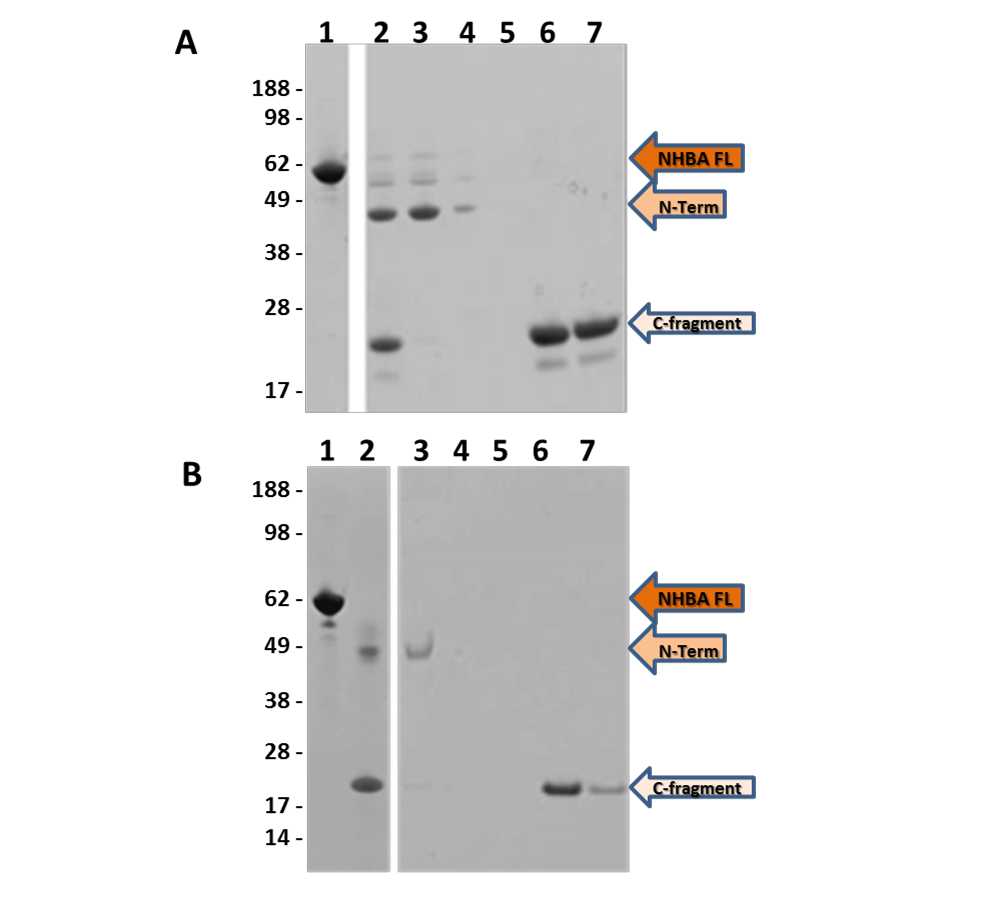

Supplement: S3 Fig — A) SDS-PAGE of C-fragment purification after NHBA processing by saliva. Lane 1: NHBA recombinant protein alone; lane 2: NHBA recombinant protein incubated with saliva overnight at 37°C; lane 3: NiNtA flow thorught fractions; lane 4 and 5: NiNtA column washes; lane 6 and 7: elution fractions from the NiNtA column which contain the C-fragment. B) SDS-PAGE of C-fragment purification from NHBA processing by hK1, in a molar ratio 1:500 between hK1 and NHBA. Lane 1: NHBA recombinant protein; lane 2: NHBA recombinant protein incubated with hK1 overnight at 37°C; lane 3: NiNtA flow thorught fractions; lane 4 and 5: NiNtA column washes; lane 6 and 7: elution fractions from the NiNtA column which contain the C-fragment. (TIF) [file pone.0203234.s003.tif]
